# Everybody's Page

**Published:** 1888-12-22

**Authors:** 


### Everybody's Page.

The Empress Eugenie has, it is said, expressed a wish that her body should be cremated after her death.

It is calculated that taking the world as a whole sixty-seven people die every minute.

In a seaport town, a floating hospital is as good as any other, and perhaps better, for the isolation of cases of infectious disease.

The colour of the eyes may change in time, and the blue-eyed damsel, one has known in youth, may change, literally, into a green-eyed monster.

The mode in which tobacco is consumed tends to lessen the poisonous effect of the nicotine, much of it being destroyed by the heat of the burning leaves before it is inhaled.

Soap makers prefer rain, river, or lake water to spring water when they can obtain it, because the former do not contain those salts of lime or magnesia which decompose the soap and waste it.

The dust-bin is a sanitary blunder, if not a sanitary crime. The method employed in the large Scotch towns of having all refuse removed daily by the municipality should be practised everywhere. Disease is promoted, if not germinated, by the storing up of possibly putrescent rubbish.

The Plague, or Black Death, as it was first called, carried away a third of the population of Europe when it appeared in the fourteenth century, and in 1665, when it came among us for the last time, it killed 68,500 of the half a-million of people who then composed the population of London.

A bill has been introduced in the Legislature of the State of New York, ordaining that no substance shall be used in embalming a dead body, which will interfere with the action of any chemical tests that might be employed in medico-legal investigations. The Americans are afraid that embalming, which is more common there than on this side of the water, may be used to conceal the action of poison.

Some time ago Dr. Axel Holst, of Christiana, inoculated a patient who was suffering from cancer with the virus of erysipelas, it having recently been suggested that the one disease was an antidote to the other. The progress of the cancerous growth was checked to a slight extent, but the patient's life was not saved, and it is probable that most people would think the cure was little better than the disease.

Aristotle devoted some attention, among his other studies, to the characteristics of ears. Large ears, according to him, indicate imbecility; flat ears belong to the coarse and brutal; small ears, dear ladies, imply bad temper and madness; and we ought all to desire firm ears of medium size. But square ears are the best of all, he who possesses them will be characterized by sublimity of soul and purity of life. This is eminently satisfactory, but what, oh what did the philosopher mean by a square ear?

A remedy that has been for some time in use, and has won golden opinions both among physicians, nurses, and ladies, is *Vinolia*, manufactured by Messrs. Roberts and Co., of New Bond Street. We have given it repeated trials, especially in cases of eczema, and in the proportion of five in every six cases have been exceedingly pleased with the results. It is emphatically what is called "an elegant preparation;" that is, it is clean, and not greasy; pleasant smelling instead of malodorous. The only thing that need be said of it is that medical men, nurses, and others should try it for themselves.

A bill has been introduced into the legislature of the State of Kentucky, forbidding marriage with an idiot, lunatic, pauper, vagrant, tramp, gambler, or felon. These restrictions are wise and reasonable, and it is just possible that human authority may be able successfully to put them in force. But the Kentuckians also want to forbid marriage to anyone characterised by a violent temper. Now both husbands and wives often declare that they did not know how disagreeable their spouses temper was till the knot there's no undoing had been tied.

The fashion of men wearing the hair long was checked in 1129 by a dream. A certain knight, says the legend, dreamed that an enemy came and strangled him with his long tresses. So impressed was he with his dream, that as soon as he awoke he cut his hair short, and told all his friends the reason why. The story went the length and breadth of the land, and all the knights in England cut their hair. But the effect of the fearful dream passed away in about a-year, and, as an indignant chronicler relates, "all who wished to appear fashionable returned to their former wickedness, and contended with the ladies in their length of hair."

While exercise is essential to most people, it seems that there are some exceptionally constituted individuals who do not require it. The Rev. William Davies, of Hereford, who died in 1790, at the age of a hundred and five, was one of these. For the last thirty-five years of his life his severest exercise was slipping one foot before the other as he went from room to room. He never went out, never even went downstairs, nor even really lifted his feet from the ground. He was a good eater, and not only took a heavy dinner, but indulged in that terror of the dyspeptic, a hot supper. Yet he never suffered from gout, paralysis, or any of the characteristic diseases of old age, but he was blind for a number of years before his death.

Dr. David Cheever, of Boston, has recently pointed out some defects in modern surgery, resulting from the use of anæsthetics. He is of opinion that the nausea resulting from anæsthetics, the temptation to try long and exhausting operations given by the unconsciousness of the patient, and the low temperature resulting from the patient getting no food for some time before the operation, and being unable to retain food for some time after, because of the nausea—all these things form a serious danger. He does not, of course, advocate the disuse of anæsthetics, but he points out that there are two sides to everything, and strongly advises that all operations should be done with as much speed as possible, that the anæsthetic should not be administered till just before the operation begins, and that every care should be taken to avoid chilling the patient.

When inoculation, the precursor of vaccination, was first intoduced into this country, it was practised by all and sundry, qualified and unqualified. It certainly requires a certain amount of courage thus to go and invite even a mild attack of small-pox, and one cannot imagine that a very strong temptation to do so was given by that amateur surgeon who announced, in the most purely phonetic spelling in an old copy of the *Oxford Journal*, that he had "inokelated these two seazons past between two and three hundred for the small-pox, and but two or three of them died." The advertiser, or George Ridler, of Stroud, adds by way of encouragement, that though "mainy peepel" dreaded inoculation, "it is no more than scrattin a bit of a haul in their yarm, a pushin' in a piece of skrapel dipt in sum of the pocky matter of a child under the distemper." No confusing technicalities in this description!

